# Impact of the primary care curriculum and its teaching formats on medical students’ perception of primary care: a cross-sectional study

**DOI:** 10.1186/s12875-016-0532-x

**Published:** 2016-09-15

**Authors:** Christopher Chung, Hubert Maisonneuve, Eva Pfarrwaller, Marie-Claude Audétat, Alain Birchmeier, Lilli Herzig, Thomas Bischoff, Johanna Sommer, Dagmar M. Haller

**Affiliations:** 1 Primary Care Unit, Faculty of Medicine, University of Geneva, Rue Michel Servet 11, Geneva 4, 1211 Switzerland; 2 Institute of Family Medicine, Faculty of Medicine, University of Lausanne, Rue du Bugnon 44, Lausanne, 1011 Switzerland

**Keywords:** Undergraduate medical education, Primary care, Career choice, Hidden curriculum, Cross-sectional, Switzerland

## Abstract

**Background:**

Switzerland is facing an impending primary care workforce crisis since almost half of all primary care physicians are expected to retire in the next decade. Only a minority of medical students choose a primary care specialty, further deepening the workforce shortage. It is therefore essential to identify ways to promote the choice of a primary care career. The aim of the present study was to explore students’ views about the undergraduate primary care teaching curriculum and different teaching formats, and to evaluate the possible impact of these views on students’ perceptions of primary care.

**Methods:**

We surveyed fifth year medical students from the Medical Faculties in Geneva and Lausanne, Switzerland (*n* = 285) with a four sections electronic questionnaire. We carried out descriptive analyses presented as frequencies for categorical data, and means and/or medians for continuous data.

**Results:**

The response rate was 43 %. Overall, primary care teaching had a positive impact on students’ image of primary care. In Lausanne, primary care curricular components were rated more positively than in Geneva. Curricular components that were not part of the primary care teaching, but were nevertheless cited by some students, were frequently perceived as having a negative impact.

**Conclusions:**

The primary care curriculum at Lausanne and Geneva Universities positively influences students’ perceptions of this discipline. However, there are shortcomings in both the structure and the content of both the primary care and hidden curriculum that may contribute to perpetuating a negative image of this specialization.

**Electronic supplementary material:**

The online version of this article (doi:10.1186/s12875-016-0532-x) contains supplementary material, which is available to authorized users.

## Background

Primary care represents the first point of entry into the healthcare system for most patients. Primary care physicians offer comprehensive and continuous care and can make efficient use of healthcare resources through appropriate coordination of care [1]. Health systems with a strong primary care basis are known to improve overall health outcomes [2]. In Switzerland, however, primary care physicians (specialised in general internal medicine and paediatrics) make up only one third of all practicing physicians [3]. The country is facing an impending primary care workforce crisis since almost half of all primary care physicians are expected to retire in the next decade [4]. Additionally, young practitioners are increasingly working part-time. Furthermore, the ageing of the population will lead to an increase in demand. By the year 2030 up to 40 % of the demand of primary care consultations may not be met [5].

Only a minority of medical students choose a primary care specialty, further deepening the workforce shortage in Switzerland [4–6], as in many European and North American countries [7, 8]. In Geneva in 2012, only around 16 % of final-year medical students planned a career in ambulatory general internal medicine or paediatrics [9]. Proportions were somewhat higher in other Swiss Faculties (Lausanne 31%, Bern 18 %, Zurich 37 %), and overall 30 to 40 % of final year students were undecided. Nevertheless, in order to avoid a future shortage of primary care physicians it is essential to identify ways to promote the choice of a primary care career. The influences on students’ career choices are complex, including personal background, the students’ environment, and post-graduate opportunities [10–14]. Experiences during medical studies influence students’ attitudes and representations about primary care [13–15]. It is likely that these perceptions of primary care as a specialty have an impact on career choice [16].

Switzerland has five university-affiliated medical schools (Geneva and Lausanne in the French-speaking part, Zurich, Bern and Basel in the German-speaking part), and each of them has an institute of primary care. All students follow a 6-year medical course, divided into a 3-year Bachelor’s degree (pre-clinical) and a 3-year Master’s degree (clinical). Academic primary care research and teaching vary between the five medical schools [17].

To date, research in Switzerland on the influence of primary care teaching mostly focuses on postgraduate education. One exception is a recent study in Geneva which showed that during the first 3 years of study, students’ image of the primary care profession deteriorated, with respect to emotional burden, job attractiveness, financial risk, income and regulation [18]. The authors’ hypothesised that this was due to the content and structure of the preclinical curriculum. A study conducted in Basel showed that a primary care tutoring program had a positive impact on students’ motivation for a career in primary care [19], confirming that the pre-graduate curriculum has an influence on students’ perceptions of primary care. We are not aware of published research from Switzerland, or other European countries, studying students’ views of the entire primary care pre-graduate curriculum and its impact on their perception of primary care.

The aim of the present study was therefore to explore students’ views about the pre-graduate primary care teaching curriculum and about different teaching formats, and to evaluate the impact of these views on students’ perceptions of primary care.

## Methods

### Participants and setting

We surveyed fifth year medical students from the Medical Faculties in Geneva and Lausanne, Switzerland. We considered that these students had a comprehensive overview of their medical studies, and that most of them (60 to 70%) would have made a decision about their postgraduate specialty training [9]. We preferred fifth year over sixth year students, as this population was more accessible, being present at medical school for classes (unlike sixth year students, who were spread over different hospitals for clerkships). In the 2013/2014 academic year, there were a total of 285 fifth year students (164 in Lausanne and 121 in Geneva). The teaching formats in Geneva and Lausanne are quite similar except for some courses and seminars (Table 1). Differences include courses focusing more on psychosocial issues in the first year in Geneva, in which some primary care physicians are involved, a 72-h course (lectures) in the fourth year in Lausanne compared to a similar number of hours in Geneva but in a seminar format, and a compulsory 4 week clerkship in primary care practices in the final year in Lausanne [20].

**Table 1:** Primary care teaching activities at the Lausanne and Geneva medical faculties [17]

	Lausanne	Geneva
Small-group seminars led by primary care physicians	40 h (1^st^ to 4^th^ year)	109 h (2^nd^ to 5^th^ year)
Lectures by primary care physicians	38 h (1^st^ to 6^th^ year) “Generalism module” (132 h in 4^th^ and 5^th^ years) ^a^	15 h (1^st^ and 2^nd^ year)
Mandatory clerkships in primary care practices	1 half-day (2^nd^ year) 4 half-days (3^rd^ year) 4 half-days (4^th^ year) 1 month (5^th^ year)	4 half-days (2^nd^ year) 8 half-days (4^th^ year)

^a^ Teaching shared by the Primary Care Unit and the University hospital-based outpatient primary care division (general medicine and emergency services)

### Survey instrument

The questionnaire was developed from November 2013 to January 2014 at the Primary Care Unit in Geneva, in collaboration with medical education specialists from the Unit of Development and Research in Medical Education in Geneva and the Institute of Family Medicine in Lausanne. We pilot-tested the survey with seven fifth year medical students from both medical schools and adapted the questions according to their feedback. An electronic version of the questionnaire was submitted in February 2014, ensuring respondents’ anonymity. Students were invited to participate via e-mail, and an oral recall was made during seminars and lectures. To encourage participation, students were offered the possibility to win a gift card (value 100 CHF) drawn among all the participants.

The final survey was made up of four sections:

1. *Students’ identification of primary care teaching and its impact on their perception of primary care:* We asked the students to list up to five curriculum components that they thought had influenced their image of primary care, and to rank this impact on a 4-point Likert scale from “very negative” to “very positive”. We preferred free-text answers rather than a pre-defined list, to encourage intuitive responses. Students were also asked to state why the chosen component had a positive or negative impact.
2. *Students’ perception of the extent to which five key aspects of primary care were covered in the curriculum:* These five key characteristics were based on the World Organisation of Family Doctors’ definition of general practice [1] and chosen following a nominal group technique consensus discussion involving medical teachers of the Primary Care Unit in Geneva (Table 2). We asked students to rate whether each of the aspects was sufficiently covered in the curriculum, on a 4-point Likert scale from “strongly disagree” to “strongly agree”.

**Table 2:** Proportion of students who agreed or strongly agreed that each of five key aspects of primary care were sufficiently covered in the curriculum

Primary care characteristics	Geneva, *n* = 54, % (95 % CI)	Lausanne, *n* = 64, % (95 % CI)
Providing comprehensive care to patients of all ages	*n* = 47, 87 % (78–96)	*n* = 54 84 % (75–93)
Psychosocial aspects	*n* = 53 98 % (95–100)	*n* = 57 89 % (81–97)
Clinical reasoning and decision analysis in an outpatient context	*n* = 34 63 % (50–75)	*n* = 25 39 % (27–51)
Managing uncertainty with ambulatory diagnostic resources	*n* = 29 54 % (40–67)	*n* = 26 41 % (29–53)
Coordinating different healthcare professionals	*n* = 32 59 % (46–72)	*n* = 42 66 % (54–77)
3. *Students’ likelihood of becoming a primary care physician:* Rated on a 4-point Likert scale form “very unlikely” to “very likely”, with an additional option for “no opinion”.
4. *Demographic information:* Age and gender.

### Statistical analyses

Free-text responses to questions about curricular components were counted (number of citations), and each citation was classified into one of four teaching format categories: (1) primary care lectures, (2) primary care seminars and small group teaching, (3) primary care clerkships, and (4) components outside of the primary care curriculum. We also asked for students’ comments on each curricular component, which were used to clarify ambiguous citations. The final decision on classification of each citation was made by consensus between two of the authors (CC and DH). We carried out descriptive analyses presented as frequencies for categorical data and means or medians for continuous data. Scores of 3 or 4 on the Likert scales were considered a positive assessment. Response rates were compared using Fisher’s exact test. The likelihood of becoming a primary care physician was compared using chi-square test.

## Results

### Response rate and participants

A total of 121 questionnaires were returned, and all of them were used in the analysis. The response rate was 46 % (56/121) in Geneva and 40 % (65/164) in Lausanne. This difference was not statistically significant (*p* = 0.28). Respondents were representative of the student population in terms of age (mean age 24 years) and gender (64 % women in Geneva, 52 % women in Lausanne).

### Identification and impact of curricular components

**Fig. 1:**
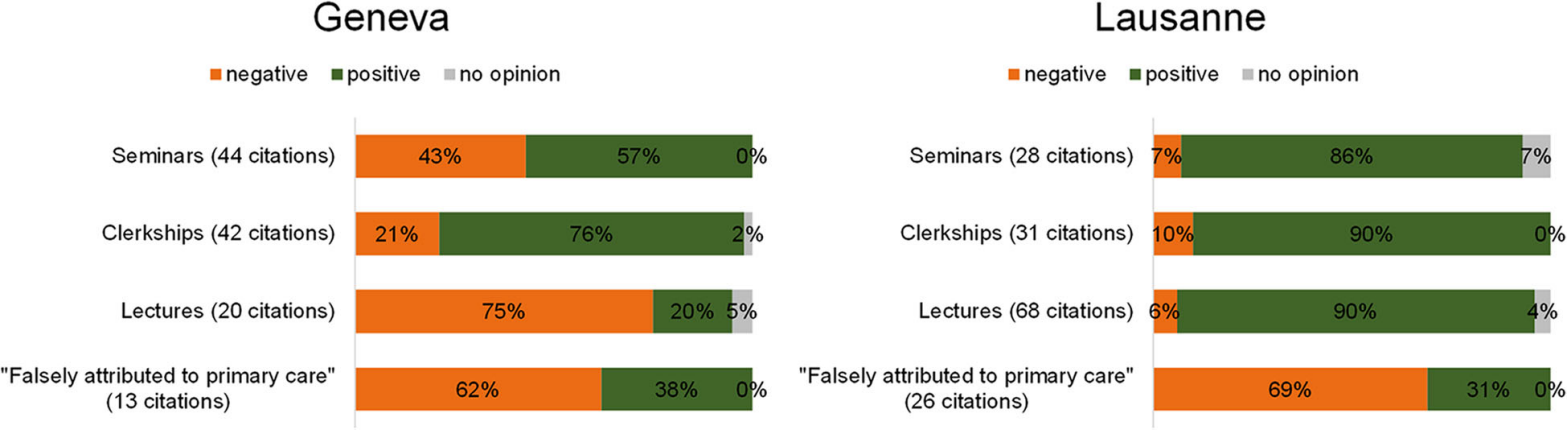
Fifth year students cited up to five primary care curricular components, and rated their impact on the image of primary care on a four-point Likert scale. A rating of 3 or 4 was considered a positive impact, a rating of 1 or 2 was considered a negative impact. The figure represents the impact of the cited components by category and by location

Students cited between 0 and 5 curricular components (total number of citations = 272, median per student = 2) that had had an impact on their image of primary care. In Geneva, the most frequently cited formats were seminars and clerkships (Fig. 1). In Lausanne, the most frequently cited components were lectures (Additional file 1). Thirty-nine citations (14 %) were curricular components not taught within the primary care curriculum (components “falsely attributed” to primary care).

Overall, the impact of primary care components was rated more positively in Lausanne than in Geneva (Fig. 1). In Geneva, lectures appeared to have a negative impact on students’ image of primary care (Additional file 2). In both sites components falsely attributed to the primary care curriculum also seemed to have a negative impact.

### Perceptions of the coverage of key characteristics

This part of the questionnaire was answered by 118 students. A majority of students perceived aspects of comprehensive care provision and psychosocial aspects to be sufficiently covered in the current curriculum (Table 2). The other three aspects (clinical reasoning, managing uncertainty and coordinating different healthcare professionals) were perceived as less well covered (Additional files 3 and 4).

### Likelihood of becoming a primary care physician

Of the 118 students who answered this question, 43 (35 %) stated that they were likely or very likely to become a primary care physician (Additional files 3 and 4). There were no significant differences between Geneva (19/54, 35 %) and Lausanne (24/64, 38 %, *p* = 0.94). The likelihood of becoming a primary care physician was not associated with the reporting of a positive or negative impact of the curriculum on the image of primary care (using chi-square test, *p* = 0.42).

## Discussion

We studied the influence of undergraduate primary care teaching components on students’ perception of primary care as a specialty at the medical faculties in Geneva and Lausanne. We found that primary care teaching overall had a positive impact on students’ image of primary care, but, in Lausanne, primary care curricular components were rated more positively than in Geneva. Curricular components that were not part of the primary care teaching, but were nevertheless cited by some students, were also frequently perceived as having a negative impact.

Our results highlight a clear difference between the two medical faculties. Students in Lausanne cited components that belonged more specifically to primary care and which appeared to have a more positive impact on students’ perception of the specialty. This difference was expected and can in part be explained by structural differences between the curricula in each site. In Lausanne, the primary care curriculum is clearly labelled “Generalism”, is more continuous, and there is a clear identification of the sessions taught by the lecturers who are part of the Institute of Family Medicine. In Geneva, the primary care curriculum is mostly taught in a seminar format, by a larger number of teachers affiliated with various structures, one from the Faculty of Medicine, and the other from the University Hospital. Longitudinal, well-structured primary care teaching programs are more susceptible to effectively increase the number of students choosing a primary care specialty [21]. We hypothesize that, in Geneva, the involvement of two units—and a multitude of teachers from private and hospital practice—leads to a less clearly structured curriculum and more difficulties for Geneva students to identify primary care teaching components. On the contrary, in Lausanne the lecture format, with a limited number of lecturers identified as family doctors and from the same institute of family medicine, allows for clearer identification. The structural differences between the two sites can be explained by the fact that Lausanne’s entire primary care curriculum was launched in 2009 as part of an educational reform favouring primary care, whereas in Geneva the primary care components were gradually added to the curriculum from the early 1990s, without a true longitudinal and global perspective.

Interestingly, a fair number of students cited curricular components that are not part of the primary care curriculum and are not taught by primary care faculty. A majority of these components were rated as having a negative impact on the image of primary care. Several hypotheses may explain these findings:

- Students may have taken the opportunity to express their general dissatisfaction with specific components (not specifically linked to their impact on the image of primary care).
- Students may have falsely attributed certain components to primary care; this may be due to an imperfect understanding of the field of primary care.
- Students may have misunderstood the question and spontaneously listed all curricular components they remembered having a negative impact on their image of primary care. For example, students cited surgery or hospital-based internal medicine clerkships as having a negative impact.

Several studies have highlighted the influence of a hidden and informal curriculum introducing a culture of misconceptions and downgrading of primary care among specialists [22–24]. Our results are consistent with these studies. They also show that a negative image of primary care can be transmitted through the formal curriculum, or at least perceived as formal by students.

Our results also show an imbalance in students’ perceptions of how different aspects of primary care are taught. Students seem to think that the psychosocial aspects of primary care and providing comprehensive care are adequately covered in class, while more technical aspects such as managing uncertainty and clinical reasoning in an outpatient context are not. Previous studies have shown that students see primary care as a specialization focused on human aspects [25]. Giving the curriculum a more coherent structure—especially by implementing a longitudinal structure within the curriculum—may help improve the image of primary care [21]. In the future it will be interesting to study the influence of introducing a stronger focus on more clinical aspects and clinical reasoning on students’ perception of primary care.

### Strengths and weaknesses

Our study’s main limitation is the response rate, which was under 50 %. The respondents’ mean age and gender were representative of the student population in the two faculties. However, we cannot exclude a selection bias, especially in the Geneva group, where the proportion of students planning a primary care career was higher than observed in previous years. Alternatively, this higher proportion could in part reflect a true increase in this career choice in relation to recent social and political focus on primary care in our country. Yet we have no recent data to confirm this. The retrospective nature of our questions may have introduced a recall bias. Due to the open-ended questioning method, students may have more willingly cited the more recent curriculum components. Nevertheless, the fact that students listed all the main primary care curricular components given over the whole study program suggests that this bias had a weak influence. The seven student pilot testers were not removed from the sample and received the questionnaire. As their answers were anonymous, we do not know whether they participated.

A more complete view of students’ perspective on the entire curriculum could have been obtained by surveying sixth year students. Due to their final year clerkships, these students are difficult to reach, and we therefore decided to limit our sample to fifth year students. Thus we cannot draw conclusions on elements that students may have encountered during their final study year. Our data were collected before the launch of the primary care education reform currently under way in Switzerland [17, 26]. Our findings thus reflect students’ views on an evolving curriculum. Yet they form a useful base for future comparisons. Replication of our study in the future may contribute to measure, to some extent, the impact of changes made to the curriculum as part of this reform.

The main strength of our study is the focus on two closely situated medical schools in the French-speaking part of Switzerland, which limited variations due to cultural differences. This also allowed us to compare and interpret our findings in light of the differences between the two curricula. However, cultural and political differences between the two regions (the urban setting of Geneva and the more rural setting of Lausanne and its surroundings) may also play a role in explaining some of our findings. Asking participants which courses they thought were related to primary care in the open-ended questions helped avoid the measurement bias that would have been introduced if we had provided them with pre-defined course lists. This method encouraged students to list curricular components that had made a strong impression on them, and unexpectedly revealed other components that seem to have had a more negative impact on their image of primary care. Thus, we were able to study the impact of the entire primary care curriculum from the first to the fifth year and its “real-life” impact on the students. In the UK, in a post-graduate junior doctor population, an association was observed between socio-economic factors and general practice choice [27]. It would be interesting to see whether these results can be replicated in an undergraduate population and in a country such as Switzerland.

The questionnaire-based format and electronic distribution method present the advantage of being easily reproducible over time and at other medical schools. It would be interesting to observe to what extent our findings can be reproduced at other medical schools in Switzerland and in other countries.

## Conclusion

Given the growing shortage of primary care physicians, understanding the factors that influence the image of primary care during undergraduate medical education is critical. The primary care curriculum positively influences students’ perceptions of this discipline, particularly in Lausanne. However, there are shortcomings in both the structure and the content of the primary care curriculum that may contribute to perpetuating a negative image of this specialization. Our findings support the previous result that providing a well-labelled longitudinal curriculum is beneficial. Further comparisons of curricula of different medical schools in Switzerland and other countries may help shed more light on this issue.

## Additional files

Additional file 1: Detailled Lausanne students’ identification of up to 5 primary care teaching and their impact on their perception of primary care on a 4-point Likert scale from 1: “very negative” to 4 “very positive”. Verbatim: description of the teaching. Classification: academic year of teaching. Impact: impact on their perception of primary care. (XLSX 17 kb) Additional file 2: Geneva sample. Detailled students’ identification of up to 5 primary care teaching and their impact on their perception of primary care on a 4-point Likert scale from 1: “very negative” to 4 “very positive”. Verbatim: description of the teaching. Classification: academic year of teaching. Impact: impact on their perception of primary care. (XLSX 16 kb) Additional file 3: Detailled Geneva students’ perception of the extent to which five key aspects of primary care were covered in the curriculum on a 4-point Likert scale from 1: “strongly disagree” to 4 “strongly agree”. Detailed Geneva students’ likelihood of becoming a primary care physician on a 4-point Likert scale from 1:”very unlikely” to 4: “very likely”, with an additional option 5: “no option”. CHAR1: Providing comprehensive care to patients of all ages, CHAR2: Psychosocial aspects, CHAR3: Clinical reasoning and decision analysis in an outpatient context, CHAR4: Managing uncertainty with ambulatory diagnostic resources, CHAR5: Coordinating different healthcare professionals, PCP: likelihood of becoming a primary care physician. (XLSX 12 kb) Additional file 4: Detailled Lausanne students’ perception of the extent to which five key aspects of primary care were covered in the curriculum on a 4-point Likert scale from 1: “strongly disagree” to 4 “strongly agree”. Detailed Lausanne students’ likelihood of becoming a primary care physician on a 4-point Likert scale from 1:”very unlikely” to 4: “very likely”, with an additional option 5: “no option”. CHAR1: Providing comprehensive care to patients of all ages, CHAR2: Psychosocial aspects, CHAR3: Clinical reasoning and decision analysis in an outpatient context, CHAR4: Managing uncertainty with ambulatory diagnostic resources, CHAR5: Coordinating different healthcare professionals, PCP: likelihood of becoming a primary care physician. (XLSX 9 kb)

## Abbreviation

CHF: Swiss Francs
